# External stimulation-controllable heat-storage ceramics

**DOI:** 10.1038/ncomms8037

**Published:** 2015-05-12

**Authors:** Hiroko Tokoro, Marie Yoshikiyo, Kenta Imoto, Asuka Namai, Tomomichi Nasu, Kosuke Nakagawa, Noriaki Ozaki, Fumiyoshi Hakoe, Kenji Tanaka, Kouji Chiba, Rie Makiura, Kosmas Prassides, Shin-ichi Ohkoshi

**Affiliations:** 1 Department of Chemistry, School of Science, The University of Tokyo, 7-3-1 Hongo, Bunkyo-ku, Tokyo 113-0033, Japan; 2 CREST, JST, K's Gobancho, 7 Gobancho, Chiyoda-ku, Tokyo 102-0076, Japan; 3 Division of Materials Science, Faculty of Pure and Applied Sciences, University of Tsukuba, 1-1-1 Tennodai, Tsukuba, 305-8577, Japan; 4 Science and Technology System Div., Ryoka Systems Inc., Tokyo Skytree East Tower, 1-1-2 Oshiage, Sumida-ku, Tokyo 131-0045, Japan; 5 Nanoscience and Nanotechnology Research Center, Osaka Prefecture University, 1-2 Gakuen-cho, Naka-ku, Osaka 599-8570, Japan; 6 PRESTO, JST, 4-8-1 Honcho, Kawaguchi 332-0012, Japan; 7 WPI-Advanced Institute for Materials Research, Tohoku University, 2-1-1 Katahira, Sendai 980-8577, Japan

## Abstract

Commonly available heat-storage materials cannot usually store the energy for a prolonged period. If a solid material could conserve the accumulated thermal energy, then its heat-storage application potential is considerably widened. Here we report a phase transition material that can conserve the latent heat energy in a wide temperature range, *T*<530 K and release the heat energy on the application of pressure. This material is stripe-type lambda-trititanium pentoxide, λ-Ti_3_O_5_, which exhibits a solid–solid phase transition to beta-trititanium pentoxide, β-Ti_3_O_5_. The pressure for conversion is extremely small, only 600 bar (60 MPa) at ambient temperature, and the accumulated heat energy is surprisingly large (230 kJ L^−1^). Conversely, the pressure-produced beta-trititanium pentoxide transforms to lambda-trititanium pentoxide by heat, light or electric current. That is, the present system exhibits pressure-and-heat, pressure-and-light and pressure-and-current reversible phase transitions. The material may be useful for heat storage, as well as in sensor and switching memory device applications.

## Abstract (web summary)

Materials that can store and release heat on demand are of use for energy storage applications. Here, the authors discover a ceramic material that can reversibly store energy from heat, light or electricity and release this energy as heat through the application of pressure.

## Graphical abstract

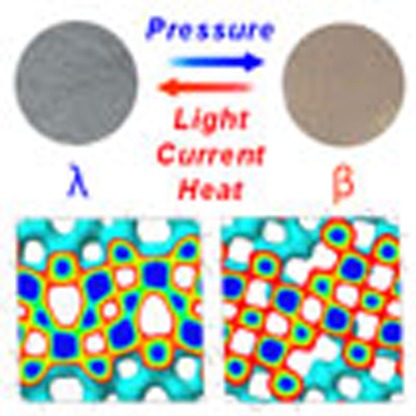

Phase transition phenomena, such as metal-insulator, ferroelectric ferromagnetic, and spin transitions, are attractive issues in the fields of physics, chemistry and materials science. Phase transitions are controlled not only by temperature change but also by other external stimuli such as pressure, light-irradiation or electric current flow. For example, for pressure-induced phase transitions, pressure-induced metal-semiconductor transition in a molybdenum disulphide^1^, pressure-induced superconductor transition in a fulleride^2^ and pressure-induced ferroelectric–antiferroelectric transition in a perovskite system^3^ have been reported. For light-induced phase transitions, light-induced crystalline-amorphous transitions in chalcogenides^4^,^5^, light-induced metal-semiconductor transition in a trititanium pentoxide^6^ and insulator-metal transition in perovskite manganites^7^,^8^, light-induced spin-crossover transitions in metal complexes^9^,^10^,^11^,^12^ and light-induced charge-transfer transition in organic molecules^13^,^14^ and metal complexes^15^ have been reported. Furthermore, for current-induced phase transitions^16^,^17^,^18^, current-induced insulator-metal transition in organic compound and current-induced magnetic-domain-wall switching in gallium manganese arsenide have been reported.

In recent years, heat-storage materials have been attracting attention from the viewpoint of energy saving. Development of high-performance heat-storage materials is important for the effective use of waste heat from blast furnaces in factories. Phase transition materials are considered to be useful as latent heat-storage materials. These are divided into solid–liquid and solid–solid phase transition types. In the former, the phase transition at the melting point (m.p.) is used for the heat storage. For example, water (320 kJ L^−1^ at m.p.=0 °C), paraffin (140 kJ L^−1^ at m.p.=64 °C)^19^ and polyethylene glycol (165 kJ L^−1^ at m.p.=20 °C)^20^ are known. In these cases, there are concerns of liquid spill from the system and mixing (or reaction) with the surrounding media. From this angle, a solid–solid phase transition material is stiff and its form is maintained without support, while at the same time it has chemical stability against the surrounding media. Well-known solid–solid phase transition materials for heat-storage usage include copolymers (for example, hyperbranched polyurethane: 150 kJ L^−1^ at 67 °C)^21^, organic compounds (for example, neopentylglycol: 165 kJ L^−1^ at 48 °C and pentaerythritol: 360 kJ L^−1^ at 188 °C)^22^,^23^ and organometallic compounds (for example, *bis*(*n*-hexadecylammonium) tetrachlorozincate: 120 kJ L^−1^ at 103 °C and *bis*(*n*-decylammonium) tetrachlorocuprate: 60 kJ L^−1^ at 34 °C)^19^,^24^,^25^. In general, such phase change heat-storage materials cannot store the energy for a prolonged period below the phase transition temperature. If a solid material could conserve the accumulated thermal energy and release it only on demand, then its heat-storage application potential is considerably widened. From this angle, our work focused on a phase transition where the latent heat of thermal phase transition could be stored.

In this paper, we report a heat-storage material composed of lambda-trititanium pentoxide. The solid–solid phase transition of this material can be controlled by heat, pressure application, light-irradiation and current flow. This heat-storage material can conserve a high accumulation of energy and release it by the application of a remarkably small external pressure.

## Results

### Material and morphology

**Figure 1:**
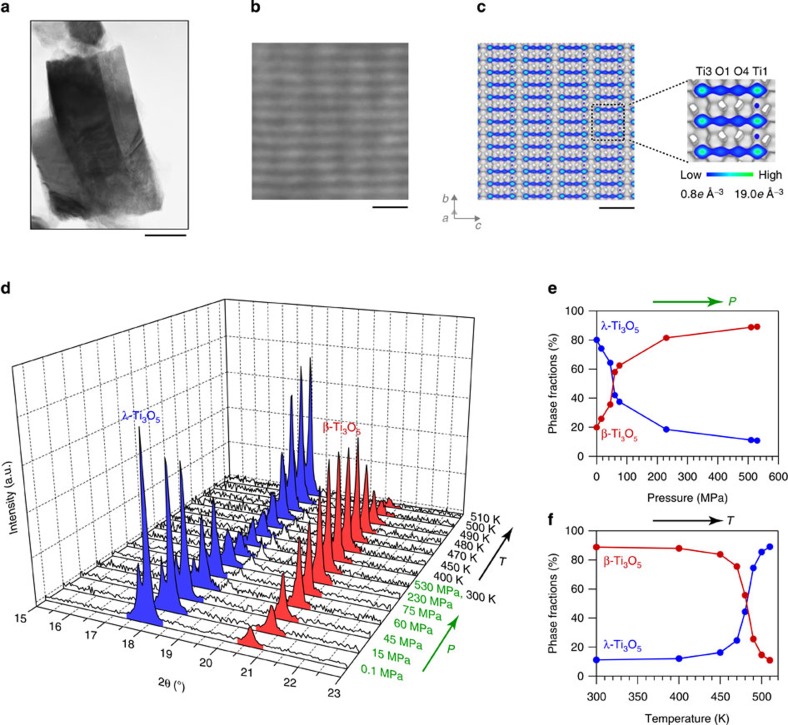
Morphology of stripe-type-λ-Ti_3_O_5_ and pressure-and-heat-induced phase transition between λ-Ti_3_O_5_ and β-Ti_3_O_5_. (**a**) TEM image of stripe-type-λ-Ti_3_O_5_. The scale bar below the TEM image indicates 50 nm. (**b**) HRTEM image of the surface of stripe-type-λ-Ti_3_O_5_ showing the atomic arrangement on the *bc* plane. The scale bar below the TEM image indicates 1 nm. (**c**) Visualized electron density maps on the *bc* plane of stripe-type-λ-Ti_3_O_5_ obtained by the MEM (isosurface 0.8*e *Å^−3^). The scale bar below the electron density map (left) indicates 1 nm. (**d**) Pressure (*P*) and temperature (*T*) dependence of the XRPD patterns (*λ*=1.5418 Å). The ambient-temperature XRPD pattern of the as-prepared sample at atmospheric pressure (*P*=0.1 MPa) is shown in the front, followed by XRPD patterns of the pellet samples pressurized by *P*=15−530 MPa, measured after pressure release. These are followed by the XRPD patterns of pressure-produced β-Ti_3_O_5_ with increasing temperature from 300 K to 510 K. (**e**) Pressure evolution of the phase fractions of λ-Ti_3_O_5_ (blue) and β-Ti_3_O_5_ (red). The pressure where the fraction of λ-Ti_3_O_5_ becomes 50% (*P*_1/2_) is an extremely small value of ∼60 MPa. (**f**) Temperature evolution of the phase fractions of λ-Ti_3_O_5_ (blue) and β-Ti_3_O_5_ (red) in the heating process.

The sample of the titanium oxide, a new series of lambda-trititanium pentoxide (λ-Ti_3_O_5_), was produced by sintering rutile-TiO_2_ particles in a hydrogen atmosphere (see Methods). Elemental analysis using inductively coupled plasma mass spectrometry confirms that the formula of the material is Ti_3_O_5_. Scanning electron microscopy (SEM) and transmission electron microscopy (TEM) images of the obtained sample show a coral-like morphology with particle size of ∼4 × 1 μm (Supplementary Fig. 1), composed of aggregates of rectangular-shaped nanorods, of which the majority are ∼200 × 30 nm dimensions (hereafter called ‘stripe-type-λ-Ti_3_O_5_', Fig. 1a). The high-resolution TEM (HRTEM) image is shown in Fig. 1b. The Fourier transform analysis of the HRTEM image showed that the growth direction of the nanorods is along the crystallographic *b* axis. The atomic level image from HRTEM corresponds to the visualized electron density distribution map on the *bc* plane calculated by the maximum entropy method (MEM; Fig. 1c), described later.

### Pressure-induced phase transition

X-ray powder diffraction (XRPD) measurements were performed to investigate the pressure (*P*) dependence of the crystal structure of the stripe-type-λ-Ti_3_O_5_. The XRPD pattern at 300 K under atmospheric pressure (*P*=0.1 MPa) is shown in Fig. 1d and Supplementary Fig. 2. Rietveld analysis indicates that this sample is composed of 80.0(2)% λ-Ti_3_O_5_ and 20.0(2)% β-Ti_3_O_5_. λ-Ti_3_O_5_ adopts a monoclinic crystal structure (space group *C*2/*m*) with lattice parameters of *a*=9.83119(19) Å, *b*=3.78798(7) Å, *c*=9.97039(19) Å and *β*=91.2909(7)˚, and a unit cell volume, *V*=371.207(12) Å^3^. λ-Ti_3_O_5_ has three symmetry-inequivalent Ti sites, Ti(1), Ti(2) and Ti(3), and five-symmetry-inequivalent O sites, O(1) to O(5). All the Ti sites form a six-coordinate structure. In the previous investigation^6^ of the same polymorph prepared from anatase-TiO_2_ nanoparticles, we observed some indications of a pressure effect. In the present research, the sample was pressed at various external pressures with a pellet press, and XRPD patterns were measured for the pellets after pressure release. With increasing *P*, the intensity of the XRPD peaks of λ-Ti_3_O_5_ decreased and those of β-Ti_3_O_5_ increased (Fig. 1d and Supplementary Fig. 3). The pressure where the fraction of λ-Ti_3_O_5_ becomes 50% (*P*_1/2_) is ∼60 MPa as shown in Fig. 1e. The crystal structure of β-Ti_3_O_5_ is monoclinic (space group *C*2/*m*; *a*=9.75252(18) Å, *b*=3.80034(6) Å, *c*=9.44413(19) Å, *β*=91.5322(10)˚ and *V*=349.902(11) Å^3^) (Supplementary Fig. 4). After pressurizing the sample and releasing the pressure at room temperature, heating the sample causes β-Ti_3_O_5_ to revert back to λ-Ti_3_O_5_ at 470 K (Fig. 1d,f and Supplementary Fig. 5a). Above 530 K, λ-Ti_3_O_5_ further transforms to α-Ti_3_O_5_. On the other hand, in the cooling process from 620 to 300 K, α-Ti_3_O_5_ returns to λ-Ti_3_O_5_ (Supplementary Fig. 5b). This λ-Ti_3_O_5_ is very stable in the wide temperature range of 0<*T*<530 K. Furthermore, when external pressure was applied to this recovered λ-Ti_3_O_5_ sample, λ-Ti_3_O_5_ exhibited again the phase transition to β-Ti_3_O_5_ (Supplementary Figs 6 and 7).

**Figure 2:**
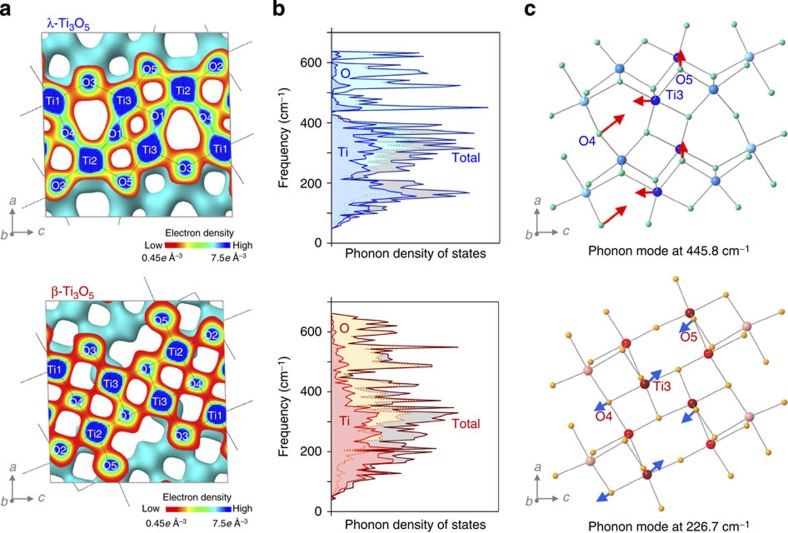
Electron density maps and the phonon modes of λ-Ti_3_O_5_ and β-Ti_3_O_5_. (**a**) Visualized electron density maps (isosurface 0.45*e* Å^−3^) of λ-Ti_3_O_5_ (upper) and β-Ti_3_O_5_ (lower) obtained using MEM from the XRPD patterns. (**b**) Phonon density of state (DOS) for λ-Ti_3_O_5_ (upper) and β-Ti_3_O_5_ (lower). Blue, light blue and grey areas indicate the contributions from phonons due to Ti, O, and the total phonon DOS, respectively for λ-Ti_3_O_5_ (upper). Red, orange and grey areas indicate the contributions from phonons due to Ti, O and the total phonon DOS, respectively, for β-Ti_3_O_5_ (lower). (**c**) Schematic illustration of the B_u_ phonon mode at 445.8 cm^−1^ for λ-Ti_3_O_5_ (upper) and the B_u_ phonon mode at 226.7 cm^−1^ for β-Ti_3_O_5_ (lower). Arrows and their lengths indicate the direction of the movement of the atoms and the relative amplitude of oscillation, respectively (see Supplementary Movies 1 and 2).

The visualized electron density distributions of λ-Ti_3_O_5_ and β-Ti_3_O_5_ obtained using MEM from the XRPD patterns, are shown in Fig. 2a. The MEM image of λ-Ti_3_O_5_ shows that the electron density is spread between both Ti and O atoms, while in β-Ti_3_O_5_, the electron density is localized around each atom. This result indicates the electron delocalized character of λ-Ti_3_O_5_ and localized character of β-Ti_3_O_5_, which are consistent with the fact that λ-Ti_3_O_5_ is a metallic conductor and β-Ti_3_O_5_ is a semiconductor. In addition, the visualized electron density distribution of λ-Ti_3_O_5_ in the *bc* plane well reproduces the HRTEM image, as mentioned in Figs 1b and c.

### First-principles calculation of phonon mode

To elucidate the pressure-induced phase transition, first-principles phonon mode calculations were conducted. Figure 2b shows the phonon density of states (DOS) based on the lattice vibrations for λ-Ti_3_O_5_ and β-Ti_3_O_5_. The phonon dispersion and phonon frequencies at the Brillouin zone centre, Γ point, for each of the phonon dispersions are listed in Supplementary Fig. 8. Comparison of the two crystal structures shows that the coordination geometry of Ti(3) is different between λ-Ti_3_O_5_ and β-Ti_3_O_5_; Ti(3) is connected to O(5) in λ-Ti_3_O_5_, while it bonds to O(4) in β-Ti_3_O_5_. Therefore, in the pressure-induced phase transition from λ-Ti_3_O_5_ to β-Ti_3_O_5_, the Ti(3)−O(5) bond is considered to break, and the Ti(3)−O(4) bond to form. The corresponding phonon modes of λ-Ti_3_O_5_ lie at 248.6, 318.5 and 445.8 cm^−1^. For example, for the B_u_ phonon mode at 445.8 cm^−1^, Ti(3) vibrates significantly toward O(4) and moves further away from O(5) (Fig. 2c (upper) and Supplementary Movie 1). On the contrary, in the course of the thermal phase transition (that is, heat-storage process) from β-Ti_3_O_5_ to λ-Ti_3_O_5_, the Ti(3)−O(4) bond is broken and the Ti(3)−O(5) bond is generated. The corresponding phonon modes now lie at 226.7 and 339.3 cm^−1^. For example, visualization of the B_u_ phonon mode at 226.7 cm^−1^ shows that Ti(3) significantly vibrates towards O(5) (Fig. 2c (lower) and Supplementary Movie 2).

### Accumulated heat energy and pressure-released energy

**Figure 3:**
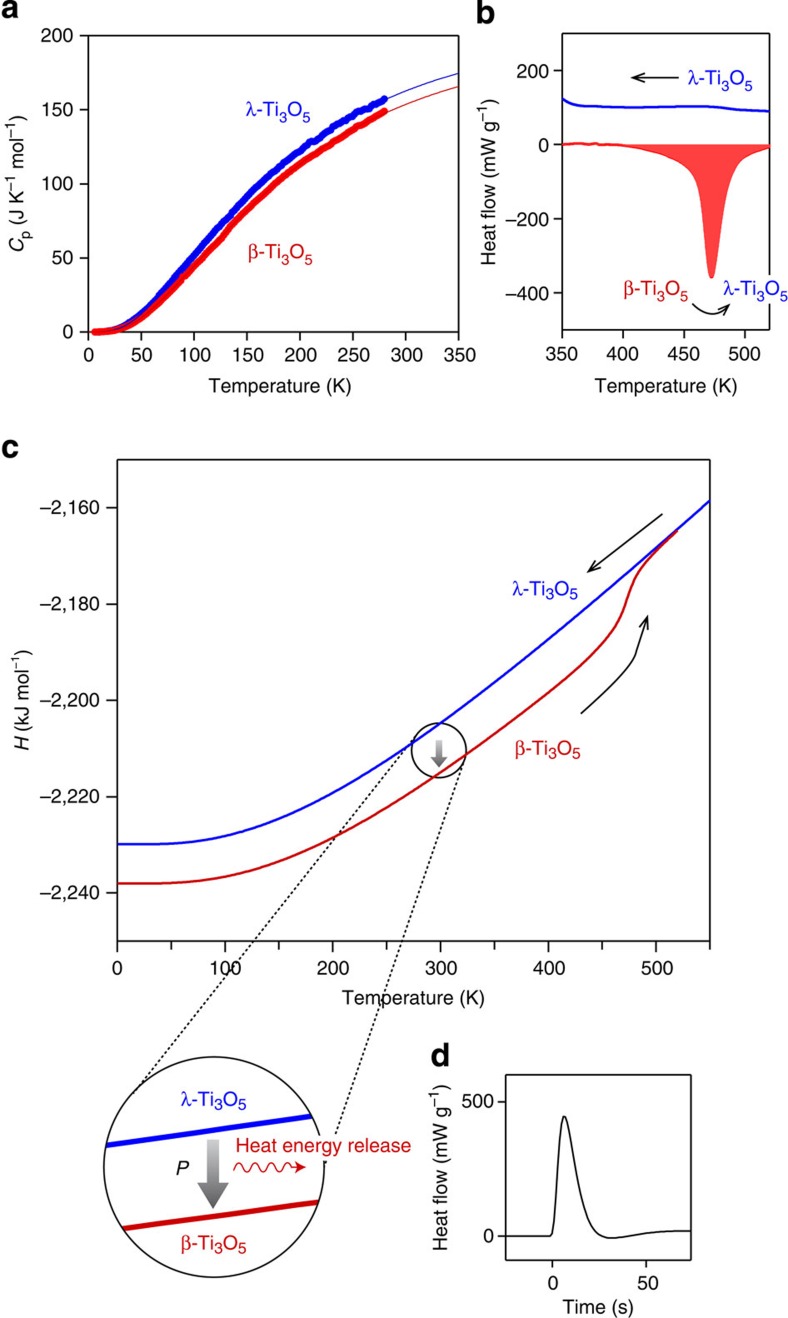
Thermodynamic properties of stripe-type-λ-Ti_3_O_5_ and pressure-produced β-Ti_3_O_5_. (**a**) Molar heat capacity of λ-Ti_3_O_5_ (blue) and β-Ti_3_O_5_ (red) as a function of temperature. Experimental data were fitted with a Debye model (see Methods). (**b**) DSC charts of the pressure-produced β-Ti_3_O_5_ with increasing temperature and λ-Ti_3_O_5_ with decreasing temperature. A peak due to the latent heat of the first-order phase transition from β-Ti_3_O_5_ to λ-Ti_3_O_5_ (230 kJ L^−1^) was observed in the heating process, whereas no peak was observed in the cooling process. (**c**) Temperature dependence of the enthalpy (*H*) for λ-Ti_3_O_5_ (blue) and β-Ti_3_O_5_ (red). When pressure is applied to λ-Ti_3_O_5_, the accumulated heat energy is released as shown in the lower enlarged figure (see Supplementary Movie 3). (**d**) Pressure-released heat energy accompanying the pressure-induced phase transition from stripe-type-λ-Ti_3_O_5_ to β-Ti_3_O_5_. Pressure was applied at *t*=0.

To investigate the heat-storage process from pressure-produced β-Ti_3_O_5_ to λ-Ti_3_O_5_ and the amount of accumulated thermal energy in the system, heat capacity measurements were performed. First, we investigated the heat capacity of the pressure-produced β-Ti_3_O_5_. In the temperature region from 5 to 300 K, specific heat was measured by the relaxation technique using the physical properties measurement system (Fig. 3a), and above 300 K, specific heat accompanying the thermal phase transition from pressure-produced β-Ti_3_O_5_ to λ-Ti_3_O_5_ was measured by differential scanning calorimetry (DSC; Fig. 3b). By combining the results from the physical properties measurement system and DSC measurements and integrating with temperature, the experimental enthalpy (*H*) curves of λ-Ti_3_O_5_ and β-Ti_3_O_5_ versus temperature were obtained up to 600 K (Fig. 3c; see Methods). The transition enthalpy (Δ*H*) associated with the first-order phase transition from β-Ti_3_O_5_ to λ-Ti_3_O_5_ was 230±20 kJ L^−1^ (12±1 kJ mol^−1^). In the temperature decreasing process of the DSC measurement, there was no peak, indicating that the accumulated heat energy of the phase transition from β-Ti_3_O_5_ to λ-Ti_3_O_5_ was conserved in the system.

Next the released energy of the pressure-induced phase transition from stripe-type-λ-Ti_3_O_5_ to β-Ti_3_O_5_ was measured using a high-pressure micro-DSC measurement system at room temperature. After applying pressure, heat energy of 240±40 kJ L^−1^ was released, which almost corresponds to the heat accumulated energy (Fig. 3d). Therefore, this material conserves the heat energy of the phase transition from pressure-produced β-Ti_3_O_5_ to λ-Ti_3_O_5_ and releases the accumulated heat energy by applying low pressure through the pressure-induced phase transition from λ-Ti_3_O_5_ to β-Ti_3_O_5_ (Supplementary Movie 3).

### Thermal conductivity and sensible heat-storage performance

Bricks and concrete are useful as sensible heat-storage materials^20^,^26^,^27^,^28^ since they release thermal energy slowly. Thermal conductivity measurements were performed for the stripe-type-λ-Ti_3_O_5_ and pressure-produced β-Ti_3_O_5_. The thermal conductivities were 0.20±0.02 W m^−1^ K^−1^ and 0.41±0.02 W m^−1^ K^−1^ for λ-Ti_3_O_5_ and β-Ti_3_O_5_, respectively, which are similar to the values of bricks (for example, 0.16 W m^−1^ K^−1^)^26^ and concrete (for example, 0.57 W m^−1^ K^−1^)^28^.

### Current-induced and light-induced phase transitions

**Figure 4:**
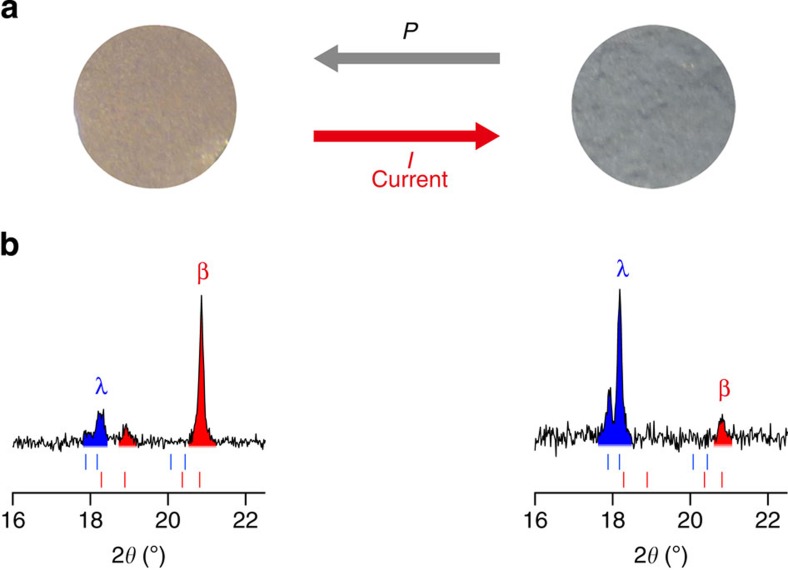
Current-induced phase transition from β-Ti_3_O_5_ to λ-Ti_3_O_5_. An electric current of 0.4 A mm^−2^ flowed through the pressure-produced β-Ti_3_O_5_ at 298 K. (**a**) Photographs of the pressure-produced β-Ti_3_O_5_ before (left) and after the application of an electric current of 0.4 A mm^−2^ (right). (**b**) XRPD pattern in the 2*θ* range of 16.0–22.5° of the pressure-produced β-Ti_3_O_5_ (left) and after the application of the electric current (right). Blue and red areas mark the peaks of λ-Ti_3_O_5_ and β-Ti_3_O_5_, respectively.

Electric current was flowed to the pressure-produced β-Ti_3_O_5_ sample at 298 K. By flowing a current of 0.4 A mm^−2^, the colour of the sample changed from brown to dark blue (Fig. 4a). The XRPD patterns before and after flowing the current indicate that β-Ti_3_O_5_ is transformed into λ-Ti_3_O_5_ (Fig. 4b and Supplementary Movie 4). The electric current dependence on the conversion from the pressure-produced β-Ti_3_O_5_ to λ-Ti_3_O_5_ shows that the threshold current value of the current-induced phase transition is 0.2 A mm^−2^ (Supplementary Fig. 9). The origin of this current-induced phase transition is regarded as breaking of charge ordering or (and) Joule heat^16^,^17^,^18^. The mechanism by breaking of charge ordering is considered as follows: β-Ti_3_O_5_ is a charge-localized state whose charge is localized on Ti^3+^(3) with empty orbital on Ti^4+^(2). In contrast, λ-Ti_3_O_5_ is a charge-delocalized state whose charge is delocalized on Ti(2) and Ti(3). By flowing electric current to β-Ti_3_O_5_, the localized charge on Ti(3) is forcedly moved to the empty orbital on of Ti(2), resulting in a transition to metallic λ-Ti_3_O_5_.

Light irradiation experiment was also conducted on a pressure-produced β-Ti_3_O_5_. The reverse phase transition from β-Ti_3_O_5_ to λ-Ti_3_O_5_ was observed by irradiation of 410-nm laser light (Supplementary Fig. 10 and Supplementary Movie 5).

## Discussion

**Figure 5:**
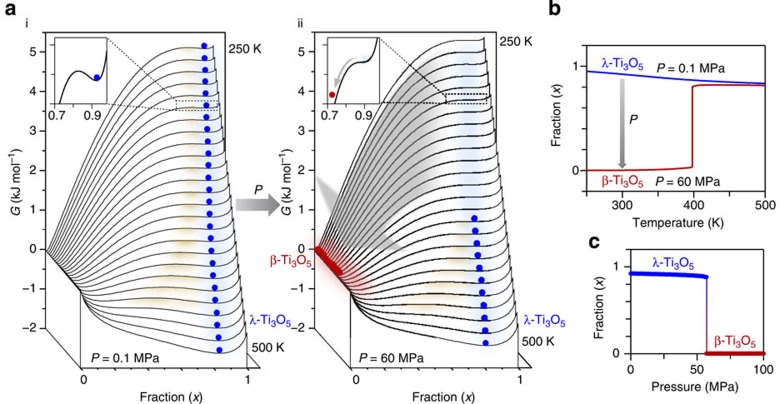
Mechanism of the pressure-induced phase transition based on a thermodynamic model. (**a**) Gibbs free energy (*G*) versus λ-Ti_3_O_5_ fraction (*x*) for every 10 K between 250 K to 500 K calculated using the Slichter–Drickamer mean-field model at *P*=0.1 MPa (i) and 60 MPa (ii). Blue and red circles indicate λ-Ti_3_O_5_ and β-Ti_3_O_5_, respectively. λ-Ti_3_O_5_ undergoes a pressure-induced phase transition to β-Ti_3_O_5_ because the energy barrier (shown by brown shadows) disappears by the application of external pressure above ∼60 MPa as shown in the insets (see Supplementary Movie 6). (**b**) Calculated *x* versus temperature curves at *P*=0.1 MPa (blue) and 60 MPa (red). (**c**) Calculated *x* versus pressure curve at 300 K indicating a threshold pressure of ∼60 MPa.

The generation of stripe-type-λ-Ti_3_O_5_ originates from the change in the Gibbs free energy (*G*) of the material compared with the bulk or single crystal Ti_3_O_5_. This change in the *G* value is considered to be due to the interface (and/or surface) energy of the nanoscale domain. It is noted that there is no oxygen vacancy, which was confirmed by electron spin resonance. To understand why the stripe-type-λ-Ti_3_O_5_ undergoes a pressure-induced phase transition to β-Ti_3_O_5_, we considered the thermodynamics of the present phase transition phenomena using the mean-field model, developed by Slichter and Drickamer^29^. In this model, *G* is described by Δ*H*, the transition entropy (Δ*S*) and the interaction parameter between λ-Ti_3_O_5_ and β-Ti_3_O_5_ phases. The calculation shows that at atmospheric pressure (*P*=0.1 MPa), the sample exists as λ-Ti_3_O_5_ (Supplementary Movie 6). This is because λ-Ti_3_O_5_ is synthesized by sintering at a high temperature, and it remains as λ-Ti_3_O_5_ with decreasing temperature due to the energy barrier between λ-Ti_3_O_5_ and β-Ti_3_O_5_ as shown in the *G* versus fraction (*x*) of λ-Ti_3_O_5_ curves (Fig. 5a (i)). On the contrary, on applying external pressure, the *G* versus *x* curves change; for example, the energy barrier disappears <400 K when *P* is 60 MPa, and hence, λ-Ti_3_O_5_ transforms into β-Ti_3_O_5_ on applying pressure (Fig. 5a (ii)). The *x* versus temperature curves of *P*=0.1 MPa and *P*=60 MPa are shown in Fig. 5b. As shown in Fig. 5c, *x* versus pressure plots indicate the threshold of the pressure-induced phase transition. The origin of the pressure-induced phase transition is the *P*Δ*V* term of Δ*H*(=Δ*U*+*P*Δ*V*), where Δ*U* and Δ*V* are the changes of internal energy and volume, respectively. At such a low pressure, the pressure-induced change on Δ*U* is very small and negligible. In fact, the phonon mode calculation under external pressure shows that the pressure-induced change of Δ*U* is ∼1 × 10^−3^ kJ mol^−1^ at 60 MPa, which is two orders smaller compared with *P*Δ*V*=0.19 kJ mol^−1^. The pressure-induced change on Δ*S* is also very small and cannot contribute to the pressure-induced phase transition in the present system (see Methods, Supplementary Fig. 11 and Supplementary Tables 1, 2). It is noted that the observed *x* versus *P* plots of Fig. 1e is somewhat gradual. This is explained by the presence of a distribution in the transition pressure of the Slichter and Drickamer model, which may be due to the crystal size distribution. We have simulated this gradual pressure-induced phase transition with a distribution of transition pressures (Supplementary Fig. 12).

In summary, we report the first metal oxide capable of conserving the accumulated heat energy of a phase transition. Stripe-type-λ-Ti_3_O_5_ can store a large heat energy of 230 kJ L^−1^, and this energy can be released by applying external pressure only when demanded. The magnitude of the required pressure is extremely small, ∼60 MPa. This value is remarkably smaller than the typical pressures observed in the pressure-induced phase transitions in metal oxide materials^30^,^31^,^32^,^33^,^34^,^35^ and metallic compounds^36^,^37^,^38^,^39^,^40^,^41^, for example, the pressure-induced phase transition from rutile-TiO_2_ to baddeleyite-type TiO_2_ at 1,043 K occurs at 20,000 MPa (=20 GPa)^30^. From the viewpoint of the energy balance of the thermodynamic cycle, pressure of 60 MPa corresponds to ∼10 kJ L^−1^, which is <5% of the pressure-releasing heat energy. Pressure of ∼60 MPa can be realized even by the water pressure of a high-pressure washing machine, and hence, λ-Ti_3_O_5_ has the potential to be employed as pressure-sensitive sheets or reusable portable heating pads. In addition, since λ-Ti_3_O_5_ is a metallic conductor and β-Ti_3_O_5_ is a semiconductor, it has possibilities as a pressure-sensitive conductivity sensor or pressure-sensitive optical sensor. Furthermore, because λ-Ti_3_O_5_ is composed of common elements (titanium and oxygen), it is safe and environmentally friendly. λ-Ti_3_O_5_ could be useful for heat-retaining systems for residential use and may realize more efficient uses of industrial waste heat generated from furnaces (Supplementary Fig. 13)^42^,^43^. In addition, light-induced and current-induced phase transitions from pressure-produced β-Ti_3_O_5_ to λ-Ti_3_O_5_ are also observed, that is, stripe-type-λ-Ti_3_O_5_ shows *reversible* pressure-and-light-induced phase transition and *reversible* pressure-and-current-induced phase transition. These effects are also attractive phenomena from the viewpoint of advanced electronic devices.

## Methods

### Material

A new series of λ-Ti_3_O_5_ nanocrystallites was produced by sintering rutile-TiO_2_ particles in a hydrogen atmosphere (flow rates of 0.7 dm^3^ min^−1^) at 1,117 °C for 2 h, followed by a slow cooling process of ∼9 h from the sintering temperature to room temperature (Supplementary Fig. 14). Elemental analysis using inductively coupled plasma mass spectrometry confirms that the formula is Ti_3.00(1)_O_5.00(6)_; Calc.: Ti, 64.2%. Found: Ti, 64.2(1)%. The experimentally obtained density is 4.000±0.048 g cm^−3^, which is consistent with the theoretical value of 4.00 g cm^−3^ from the crystal structure of λ-Ti_3_O_5_ as determined by XRPD measurements. SEM and TEM images of the obtained sample show a coral-like morphology with particle size of ∼4 × 1 μm, composed of rectangular-shaped nanorods, of which the majority are ∼200 × 30 nm dimensions (Supplementary Fig. 1a). The Fourier transform analysis of the HRTEM image showed that the growth direction of the nanorods is along the crystallographic *b* axis. This new series of λ-Ti_3_O_5_ have larger crystal size than the previous series, which were prepared from anatase-TiO_2_ (ref. 6; Supplementary Fig. 1b).

### XRPD measurements

XRPD measurements were performed with a Rigaku Ultima IV diffractometer with Cu *K*_*α*_ radiation (*λ*= 1.5418 Å). The temperature-dependent XRPD measurements were undertaken using a high-temperature chamber with atmosphere control (RIGAKU-OAT003S) under N_2_ flow. The RIETAN-FP computer programme was used for the Rietveld analyses, while Dysnomia was used for the MEM analyses. The refined crystal structures and charge densities were visualized by the computer programme VESTA. Although both λ-Ti_3_O_5_^6^ and its high-temperature phase^44^,^45^ can be considered as candidates of the present material with *C*2/*m* crystal structure, we assigned the present material to λ-Ti_3_O_5_ because it is obtained by a very slow cooling process taking of *ca*. 9 h from the sintering temperature to room temperature, and it is thermally stable.

### Heat capacity measurements

To investigate the temperature dependence of the lattice specific heat, *C*(*T*), in the temperature range of 5–300 K, we carried out curve fitting of the observed plots with the equation based on the two-Debye model^46^ expressed by 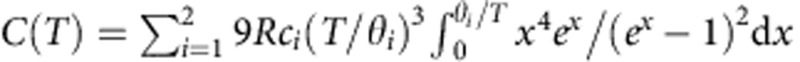, where *R* is gas constant, *c*_*i*_ is coefficient, *θ*_*i*_ is Debye temperature, *x* is 
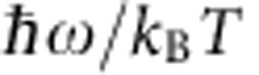
, *ħ* is the reduced Planck constant, *ω* is phonon frequency and *k*_B_ is Boltzmann constant, with the fit parameters of *c*_1_=3.2(1), *c*_2_=5.6(1), *θ*_1_=4.1(1) × 10^2^ K and *θ*_2_=9.3(1) × 10^2^ K for λ-Ti_3_O_5_, and *c*_1_=2.7(1), *c*_2_=5.8(1), *θ*_1_=4.3(1) × 10^2^ K and *θ*_2_=9.3(2) × 10^2^ K for β-Ti_3_O_5_. We then developed the temperature dependence curve of the specific heat in the temperature range of 5–600 K using both the fitted curve and the anomalous specific heat associated with the first-order phase transition from β-Ti_3_O_5_ to λ-Ti_3_O_5_ obtained from the DSC measurement.

### Released heat energy on pressure application

Released heat energy on pressure application was measured with a high-pressure DSC measurement system (μDSC VII, SETARAM Instrumentation) at 300 K. Pressure application of 40 MPa was achieved by instant injection of N_2_ gas into the sample cell.

### Thermal conductivity measurements

The specific heat and thermal diffusivity of λ-Ti_3_O_5_ and β-Ti_3_O_5_ pellet samples were measured with a DSC measurement system (DSC200F3 Maia (NETZSCH), NSST Co., Ltd.) and Light Flash Apparatus (LFA447NanoFlash, NSST Co., Ltd.), respectively.

### First-principles phonon mode calculations

First-principles calculations based on the density functional theory were carried out for λ-Ti_3_O_5_ and β-Ti_3_O_5_ using the VASP (Vienna *ab initio* simulation package) code. The wavefunctions based on plane waves and potentials of the core orbitals were represented by the projector-augmented wave of Blöchl, and the exchange-correlation term was evaluated by the generalized gradient approximation by Perdew, Burke, and Ernzerhof. The crystal structures of λ-Ti_3_O_5_ and β-Ti_3_O_5_ obtained from the XRPD measurements were used for computed models as the initial structures. The lattice parameters and atomic positions were optimized under no pressure and 1000 MPa with an energy cutoff of 500 eV and 3 × 7 × 3 *k*-mesh until satisfying 10^−5^ eV pm^−1^ force tolerance. Supercells (1 × 3 × 1) of the optimized structures were used to calculate the phonon modes and thermodynamic functions of λ-Ti_3_O_5_ and β-Ti_3_O_5_, which were calculated by the direct method implemented in Phonon code with 2 pm displacements using the optimized structures.

### Thermodynamic analysis

In the Slichter and Drickamer mean-field model, the Gibbs free energy of the system is described as *G=x*(Δ*H*)+*γx*(1−*x*)+*T*{*R*[*x* ln*x*+(1−*x*)ln(1−*x*)]−*x*(Δ*S*)}+*G*_β_, where *x* is the ratio of the charge-delocalized unit of Ti(1)^3.3+^−Ti(2)^3.3+^−Ti(3)^3.3+^ corresponding to λ-Ti_3_O_5_, *γ* is the interaction parameter between λ-Ti_3_O_5_ and β-Ti_3_O_5_ phases, *G*_β_ is Gibbs free energy of β-Ti_3_O_5_ set as the origin of the energies, and *R* is the gas constant. The observed phase transition was considered to be a metal-semiconductor phase transition between charge-delocalized Ti(1)^3.3+^−Ti(2)^3.3+^−Ti(3)^3.3+^ and charge-localized Ti(1)^3.0+^−Ti(2)^3.7+^−Ti(3)^3.3+^ systems, which were regarded as λ-Ti_3_O_5_ and β-Ti_3_O_5_, respectively. The values of Δ*H*=11.5 kJ mol^−1^ and Δ*S*=25.2 J K^−1^ mol^−1^, and a suitable value of *γ=γ*_a_+*γ*_b_ *f*(*T* ), where *γ*_a_=14 kJ mol^−1^ and *γ*_b_=1.08 × 10^−2^ J K^−1^ mol^−1^ to be consistent with the observation results, were used. When the external pressure is applied to the sample, Δ*H* is perturbed by the pressure-induced change on the Δ*U* and *P*Δ*V* terms. Compared with the pressure-induced change on the *P*Δ*V* term, for example, 0.19 kJ mol^−1^ at *P*=60 MPa, the change on Δ*U* evaluated by the first-principles phonon mode calculations is negligibly small, for example, 1 × 10^−3^ kJ mol^−1^ at *P*=60 MPa. Thus, Δ*H* is controlled by the *P*Δ*V* term in the present system. The pressure-induced change on Δ*S* is also very small, for example, −0.067 J K^−1^ mol^−1^ at *P*=60 MPa, from the results of first-principles phonon mode calculations.

### Current-induced phase transition study

Stainless electrodes are attached to β-Ti_3_O_5_ pellet by Ag paste with an adhesion area of 5 mm^2^ and electric current of 2 A was flowed (0.4 A mm^−2^) at 298 K. After that, the XRPD pattern of the surface of the pellet was measured.

## Author contributions

S.O. designed and coordinated this study, contributed to all the measurements and calculations and wrote the paper. H.T. conducted the temperature-dependent XRPD measurements, heat capacity measurements and thermodynamic calculations. M.Y. analysed the first-principles phonon mode calculation results and partially wrote the paper. K.I. carried out the Rietveld analyses of the XRPD patterns and heat capacity data analyses. A.N. conducted the Rietveld analyses and prepared the figures. T.N. carried out the pressure- and temperature-dependent XRPD measurements. K.N. carried out the elemental analyses and background research. N.O. carried out the DSC measurements and MEM analyses. F.H. obtained the TEM and SEM images. K.T. contributed to the synthesis and Rietveld analyses. K.C. carried out the first-principles phonon mode calculations. R.M. and K.P. contributed to the interpretation of the results and to the writing of the paper. All the authors commented on the manuscript.

## Additional information

**How to cite this article:** H Tokoro *et al*. External stimulation-controllable heat storage ceramics. *Nat. Commun*. 6:7037 doi: 10.1038/ncomms8037 (2015).

## Supplementary Material

Supplementary Figures and Supplementary Tables Supplementary Figures 1-14 and Supplementary Tables 1-2

Supplementary Movie 1 B_u_ phonon mode of λ-Ti_3_O_5_ corresponding to the atomic movement of the pressure-induced transformation from stripe-type-λ-Ti_3_O_5_ to β-Ti_3_O_5_. This movie shows the B_u_ phonon mode of λ-Ti_3_O_5_ at 445.8 cm^−1^ corresponding to the movement of Ti and O atoms in the pressure-induced phase transition from λ-Ti_3_O_5_ to β-Ti_3_O_5_. Ti(3) moves away from O(5) and vibrates significantly toward O(4), suggesting breaking of the Ti(3)-O(5) bond and formation of the Ti(3)-O(4) bond. Light blue and blue balls represent Ti, and gray and ocher balls represent O. The oscillation amplitude is emphasized by two times for easier viewing.

Supplementary Movie 2 B_u_ phonon mode of β-Ti_3_O_5_ corresponding to the atomic movement of the heat-induced phase transition from pressure-produced β-Ti_3_O_5_ to λ-Ti_3_O_5_. This movie shows the B_u_ phonon mode of β-Ti_3_O_5_ at 226.7 cm^−1^ corresponding to the movement of Ti and O atoms in the heat-induced phase transition from β-Ti_3_O_5_ to λ-Ti_3_O_5_. Ti(3) moves away from O(4) and vibrates significantly toward O(5), suggesting breaking of the Ti(3)-O(4) bond and formation of the Ti(3)-O(5) bond. Light pink and red balls represent Ti, and gray and ocher balls represent O. The oscillation amplitude is emphasized by two times for easier viewing.

Supplementary Movie 3 Pressure-induced phase transition from stripe-type-λ-Ti_3_O_5_ to β-Ti_3_O_5_ and heat-induced phase transition from pressure-produced β-Ti_3_O_5_ to λ-Ti_3_O_5_. The initial part of this movie displays the pressure-induced phase transition from stripe-type-λ-Ti_3_O_5_ to β-Ti_3_O_5_, where the accumulated heat energy of stripe-type-λ-Ti_3_O_5_ is released (energy release process). In the next part, the pressure-produced β-Ti_3_O_5_ is heated and transforms to λ-Ti_3_O_5_, i.e., the heat-induced phase transition accompanying accumulation of heat energy (heat storage process). The release and accumulation of heat energy are repeatedly caused by alternating application of pressure and heat. Such a metal oxide is the first example.

Supplementary Movie 4 Reversible pressure- and current-induced phase transition. This movie shows the pressure-induced phase transition from λ-Ti_3_O_5_ to β-Ti_3_O_5_ and current-induced phase transition from β-Ti_3_O_5_ to λ-Ti_3_O_5_. The initial part of this movie displays the pressure-induced phase transition from λ-Ti_3_O_5_ to β-Ti_3_O_5_, where the accumulated heat energy in λ-Ti_3_O_5_ is released (energy release process). Next, electric current is flowed to the pressure-produced β-Ti_3_O_5_ and β-Ti_3_O_5_ transforms to λ-Ti_3_O_5_, i.e., current-induced phase transition accompanying accumulation of heat energy (heat storage process). The release and accumulation of heat energy are caused repeatedly by alternating pressure application and current flow.

Supplementary Movie 5 Reversible pressure- and light-induced phase transition. This movie shows the pressure-induced phase transition from λ-Ti_3_O_5_ to β-Ti_3_O_5_ and light-induced phase transition from β-Ti_3_O_5_ to λ-Ti_3_O_5_. The initial part of this movie displays the pressure-induced phase transition from λ-Ti_3_O_5_ to β-Ti_3_O_5_, where the accumulated heat energy in λ-Ti_3_O_5_ is released (energy release process). Next, the pressure-produced β-Ti_3_O_5_ is irradiated with light and transforms to λ-Ti_3_O_5_, i.e., light-induced phase transition accompanying accumulation of heat energy (heat storage process). The release and accumulation of heat energy are caused repeatedly by alternating pressure application and light irradiation. Such a metal oxide is the first example.

Supplementary Movie 6 Mechanism of pressure-induced phase transition from stripe-type-λ-Ti_3_O_5_ to β-Ti_3_O_5_. This movie shows the mechanism of pressure-induced phase transition using the thermodynamic model proposed by Slichter and Drickamer. Gibbs free energy (*G*) versus λ-Ti_3_O_5_ fraction (*x*) curves show that λ-Ti_3_O_5_ phase (blue) is maintained at all temperatures due to the energy barrier originated from cooperative interaction between the two phases. Then, the 300 K curve is focused, showing its pressure dependence. At 60 MPa, the energy barrier disappears, and λ-Ti_3_O_5_ phase converts into β-Ti_3_O_5_ phase (red). Finally, heat storage process from β-Ti_3_O_5_ to λ-Ti_3_O_5_ is shown with *G* versus *x* and *x* versus temperature curves. Such a metal oxide is the first example.
