# Supplementary material for: External stimulation-controllable heat-storage ceramics
Source: Nat Commun. 2015 May 12;6:7037. doi: 10.1038/ncomms8037 (PMC4432584; doi:10.1038/ncomms8037)
Supplement: Supplementary Figures and Supplementary Tables — Supplementary Figures 1-14 and Supplementary Tables 1-2 [file ncomms8037-s1.pdf]

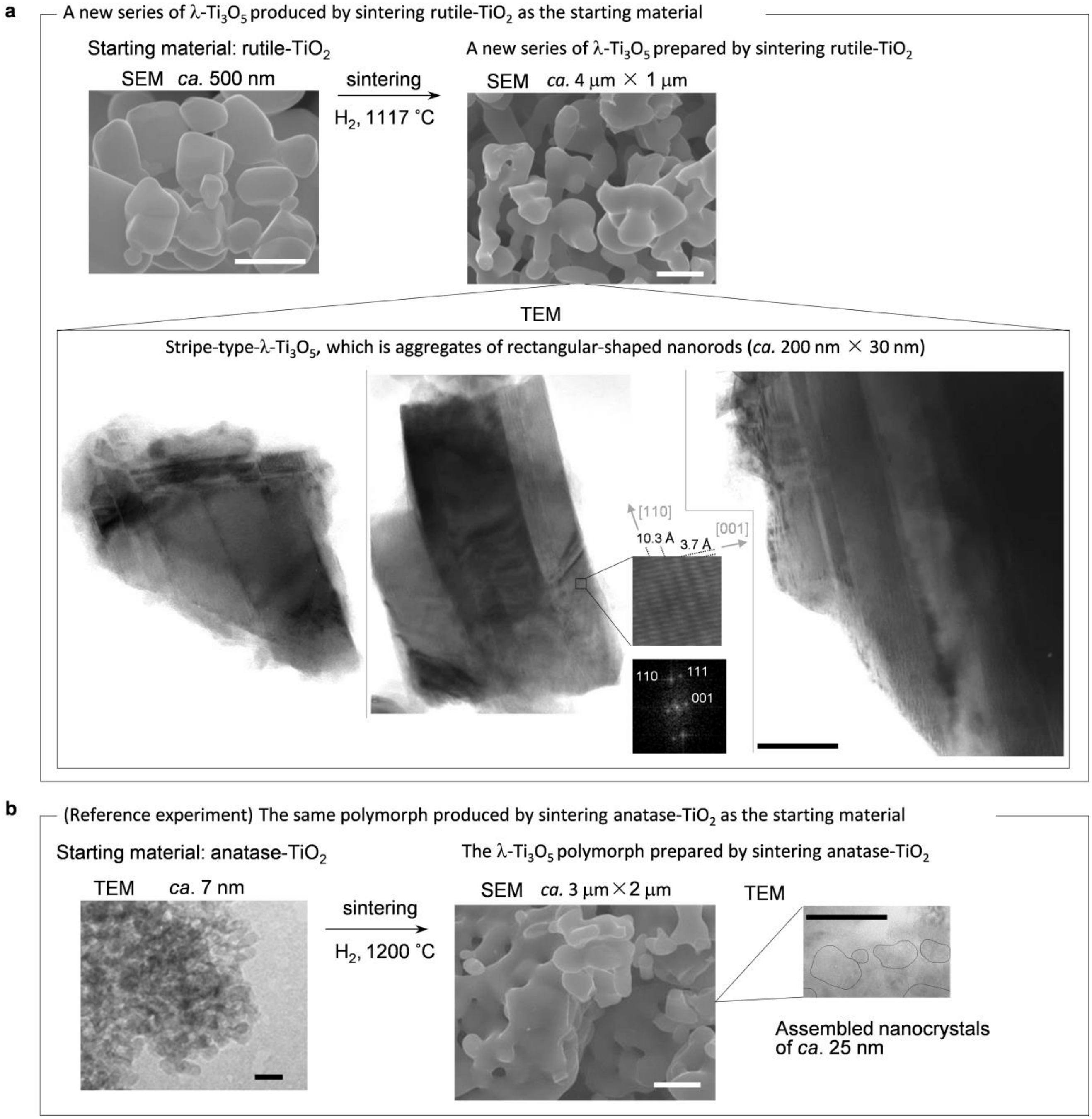

Supplementary Figure 1. A new series of stripe-type-λ-Ti_3O_5 produced by sintering rutile-TiO_2. (**a**) The present synthetic method of λ-Ti_3O_5 using rutile-TiO_2 particles. By using rutile-TiO_2 particles with a size of *ca*. 500 nm (upper left SEM image) as the starting material, λ-Ti_3O_5 with a size of *ca*. 4 μm×1 μm was obtained (upper right SEM image). The scale bars on the left and right SEM images indicate 500 nm and 2 μm, respectively. The TEM images and their Fourier transform images show that λ-Ti_3O_5 is assembled from rectangular nanocrystals of *ca*. 200 nm×30 nm and the long axis of the nanorods corresponds to the crystallographic *b* axis (hereafter called "stripe-type-λ-Ti_3O_5"). The scale bar of the TEM images indicate 50 nm. (**b**) The reference experiment of the same polymorph prepared from anatase-TiO_2 nanoparticles. By using anatase-TiO_2 nanoparticles with a size of *ca*. 7 nm (left TEM image) as the starting material, the same polymorph was obtained as particles of *ca*. 3 μm×2 μm, composed of nanocrystals of *ca*. 25 nm, as shown in the right SEM and TEM images. The reference sample was prepared as reported in Ref. 6. The scale bars on the left TEM, center SEM, and right TEM images indicate 10 nm, 2 μm, and 50 nm, respectively.

a

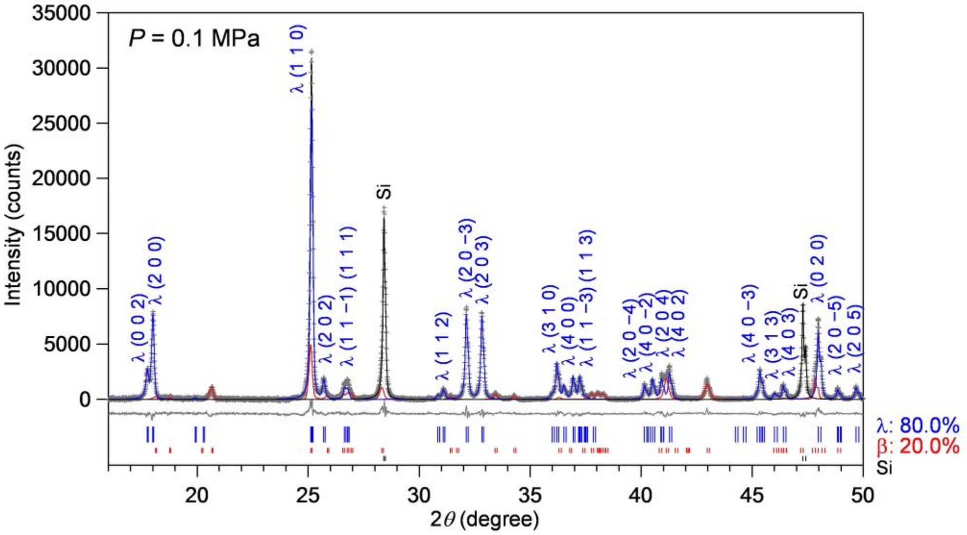

b

Polymorph		λ-Ti_3O_5	
Crystal system		Monoclinic	
Space group		*C*2/*m* (No. 12)	
a (Å)		9.83119 (19)	
b (Å)		3.78798 (7)	
c (Å)		9.97039 (19)	
β (°)		91.2909 (7)	
V (Å^3)		371.207 (12)	
Z		4	
R_{wp} (%)		3.30	
S		1.52	
	x/*a*	*y*/*b*	*z*/*c*
Ti(1)	0.6292 (2)	0	0.0531 (2)
Ti(2)	0.30586 (14)	0	0.2460 (2)
Ti(3)	0.6330 (2)	0	0.4341 (2)
O(1)	0.4561 (6)	0	0.3884 (4)
O(2)	0.1778 (5)	0	0.0667 (5)
O(3)	0.7321 (5)	0	0.2492 (5)
O(4)	0.5421 (5)	0	0.8722 (5)
O(5)	0.1908 (5)	0	0.4265 (5)

Supplementary Figure 2. XRPD pattern, Rietveld analysis, and refined structural parameters for stripe-type-λ-Ti_3O_5. (**a**) XRPD pattern (λ = 1.5418 Å) and Rietveld analysis of stripe-type-λ-Ti_3O_5 produced by sintering rutile-TiO_2. Gray plots, blue line, red line, black line, and gray line are the observed pattern, calculated pattern of λ-Ti_3O_5, calculated pattern of β-Ti_3O_5, total calculated pattern, and the residual pattern, respectively. Blue, red, and black bars represent the calculated positions of the Bragg reflections of λ-Ti_3O_5, β-Ti_3O_5, and silicon standard, respectively. The XRPD pattern was collected at room temperature under atmospheric pressure (0.1 MPa). (**b**) Structural parameters derived from Rietveld analysis of the XRPD pattern of λ-Ti_3O_5.

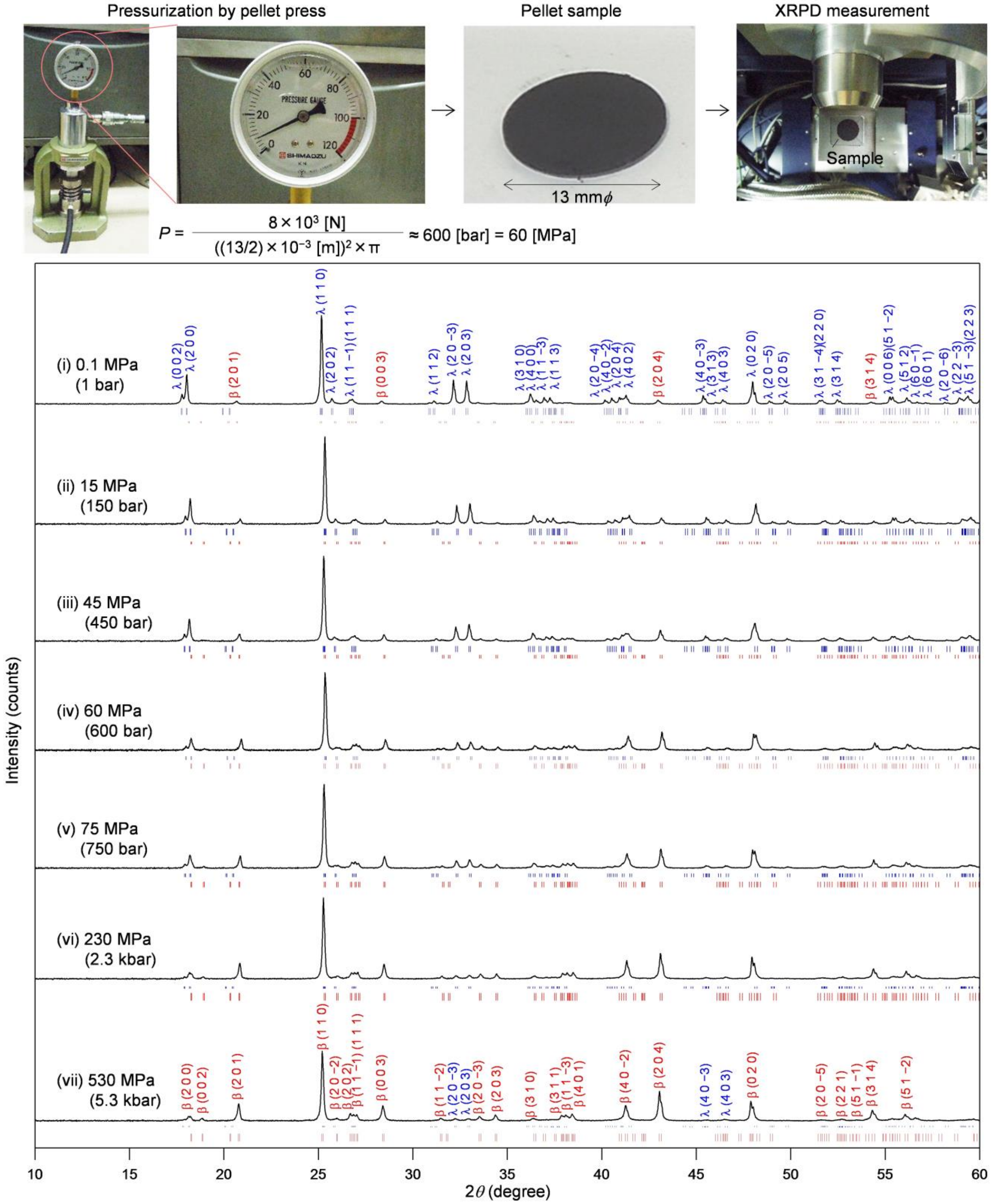

Supplementary Figure 3. Pressure dependence of the XRPD patterns measured at room temperature. The upper photographs show the experimental process of pressurization by a pellet press and XRPD measurement of the pellet.
(i) corresponds to the XRPD pattern of the sample under atmospheric pressure (0.1 MPa), and (ii)-(vii) show the XRPD patterns after applying pressure.

a

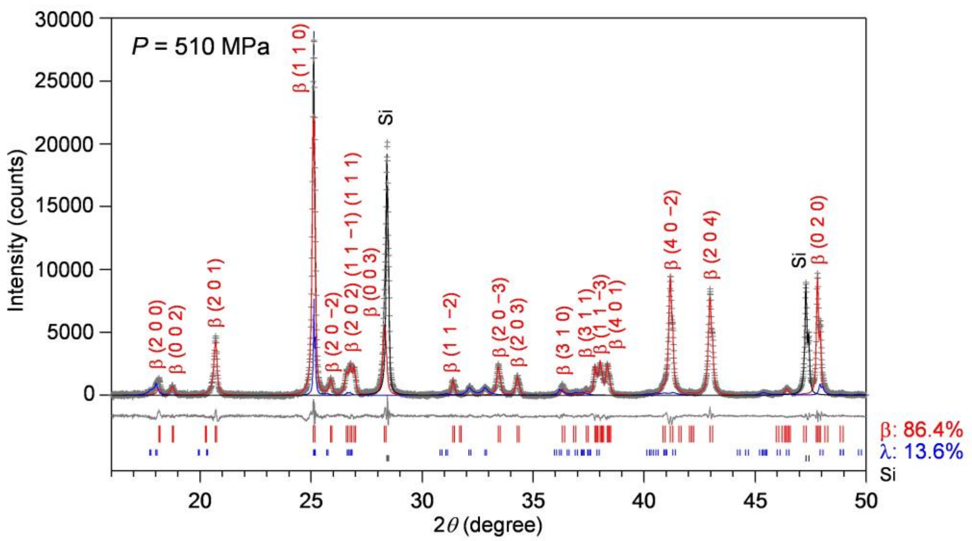

b

Polymorph		β-Ti_3O_5	
Crystal system		Monoclinic	
Space group		*C*2/*m* (No. 12)	
a (Å)		9.75252 (18)	
b (Å)		3.80034 (6)	
c (Å)		9.44413 (19)	
β (º)		91.5322 (10)	
V ($Å^3$)		349.902 (11)	
Z		4	
R_{wp} (%)		3.48	
S		1.63	
	x/*a*	*y*/*b*	*z*/*c*
Ti(1)	0.1272 (2)	0	0.0445 (3)
Ti(2)	0.7795 (2)	0	0.2673 (2)
Ti(3)	0.0545 (2)	0	0.3635 (2)
O(1)	0.6717 (8)	0	0.0586 (8)
O(2)	0.2352 (7)	0	0.2447 (8)
O(3)	0.5930 (6)	0	0.3473 (7)
O(4)	0.9516 (7)	0	0.1545 (8)
O(5)	0.8728 (6)	0	0.4334 (8)

Supplementary Figure 4. XRPD pattern, Rietveld analysis, and refined structural parameters of pressure-produced β-Ti_3O_5 obtained by applying external pressure to stripe-type-λ-Ti_3O_5. (**a**) XRPD pattern (λ = 1.5418 Å) and Rietveld analysis of pressure-produced β-Ti_3O_5 obtained by applying external pressure of 510 MPa to λ-Ti_3O_5 and then releasing the pressure. Gray plots, red line, blue line, black line, and gray line are the observed pattern, calculated pattern of β-Ti_3O_5, calculated pattern of λ-Ti_3O_5, total calculated pattern, and the residual pattern, respectively. Red, blue, and black bars represent the calculated positions of the Bragg reflections of β-Ti_3O_5, λ-Ti_3O_5, and silicon standard, respectively. The XRPD pattern was collected at room temperature under atmospheric pressure (0.1 MPa). (**b**) Structural parameters derived from Rietveld analysis of the XRPD pattern of pressure-produced β-Ti_3O_5.

a

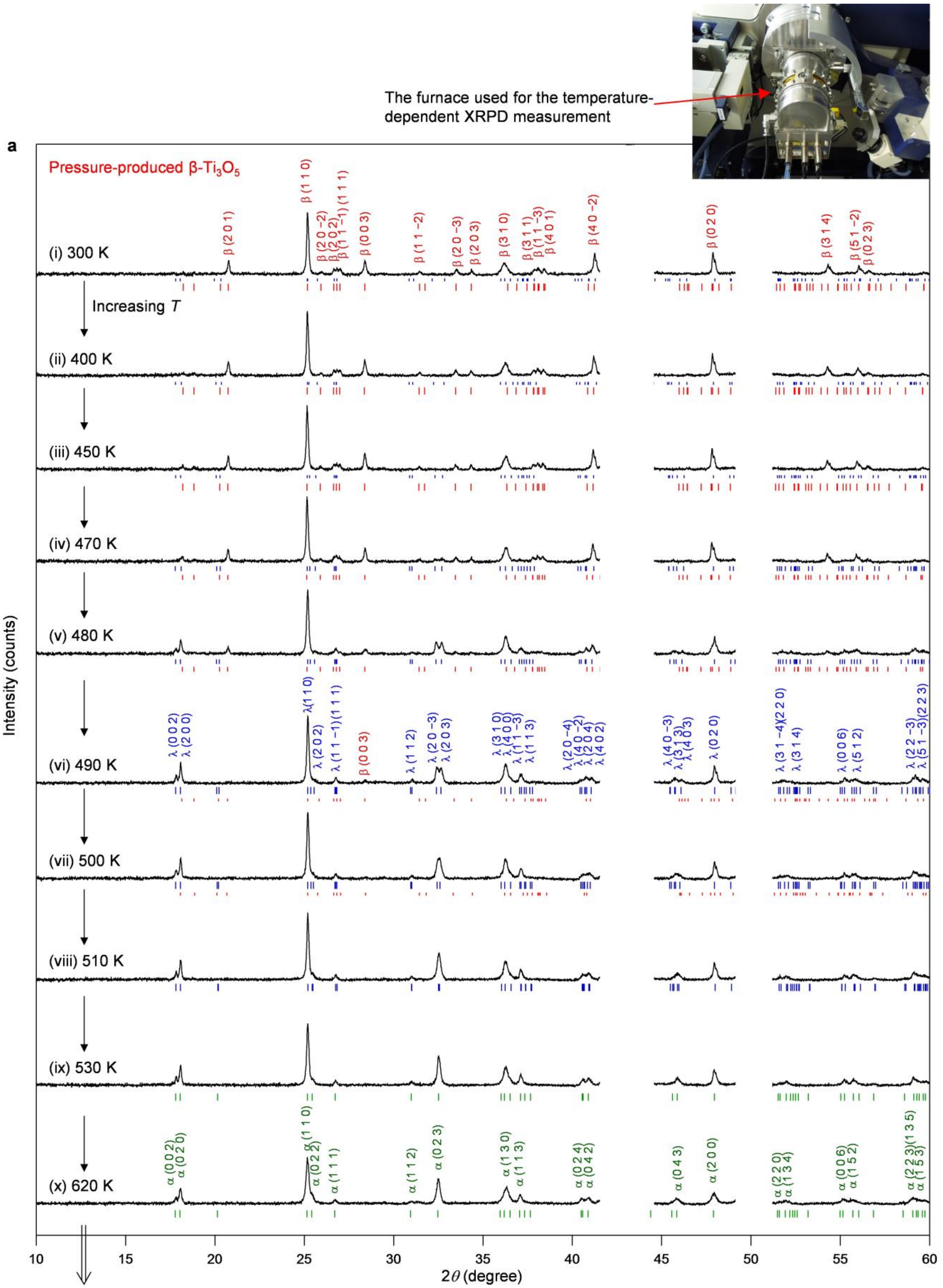

Continues to the next page.

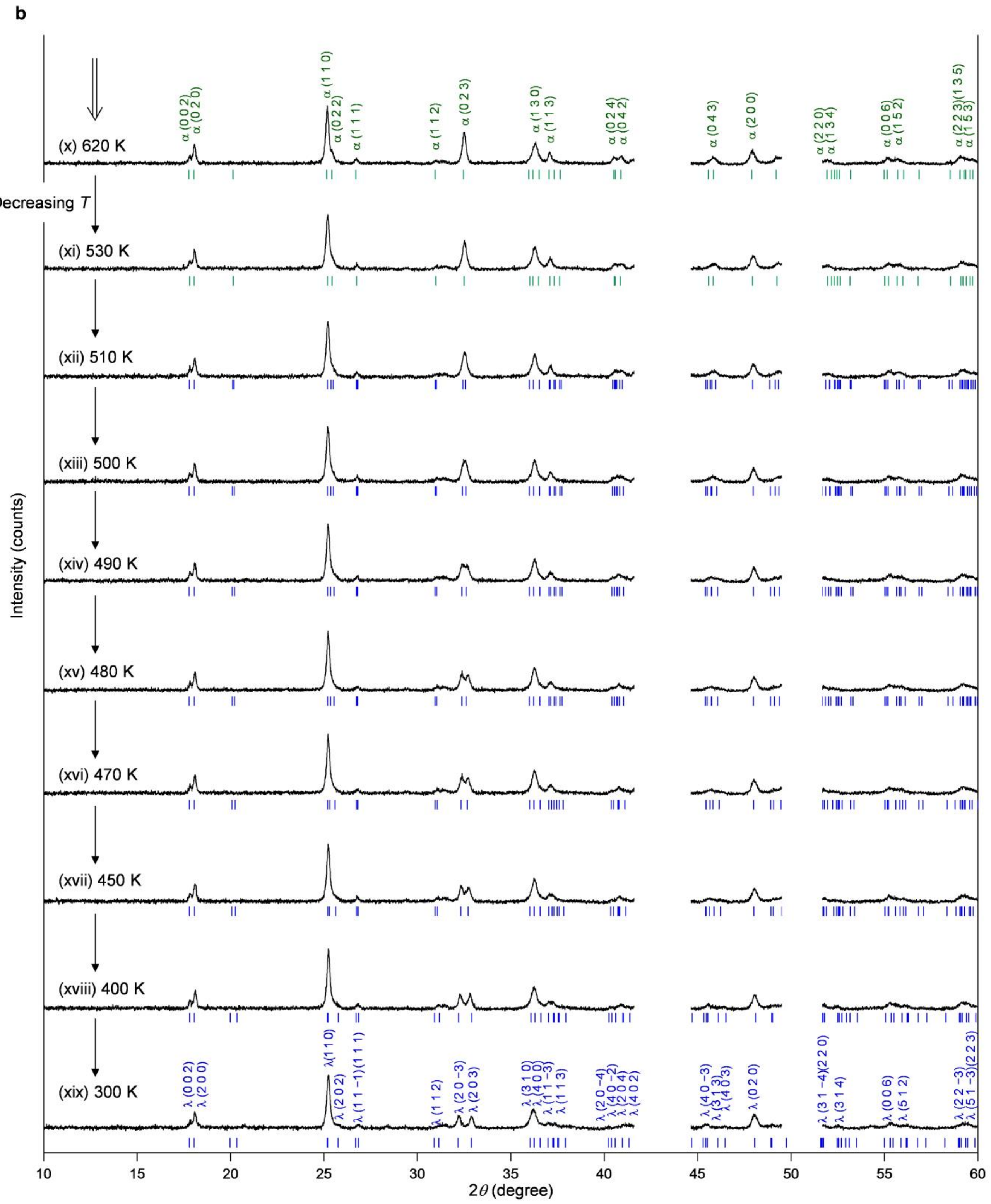

Supplementary Figure 5. Temperature dependence of the XRPD patterns of the pressure-produced β-Ti_3O_5. Pressure-produced β-Ti_3O_5 was prepared by applying pressure of 510 MPa and then releasing the pressure. XRPD measurements were performed on the pressure released sample at (i) 300 K, (ii) 400 K, (iii) 450 K, (iv) 470 K, 480 K, (vi) 490 K, (vii) 500 K, (viii) 510 K, (ix) 530 K, and (x) 620 K by increasing temperature (**a**), and (x) 620 K, (xi) 530 K, (xii) 510 K, (xiii) 500 K, (xiv) 490 K, (xv) 480 K, (xvi) 470 K, (xvii) 450 K, (xviii) 400 K, and (xix) 300 K by decreasing temperature (**b**). The diffraction peaks from the sample holder of the heating apparatus are excluded. The blue, red, and green tick marks are the Bragg peak positions for λ-Ti_3O_5, β-Ti_3O_5, and α-Ti_3O_5, respectively, and the length of the marks denote the phase fractions.

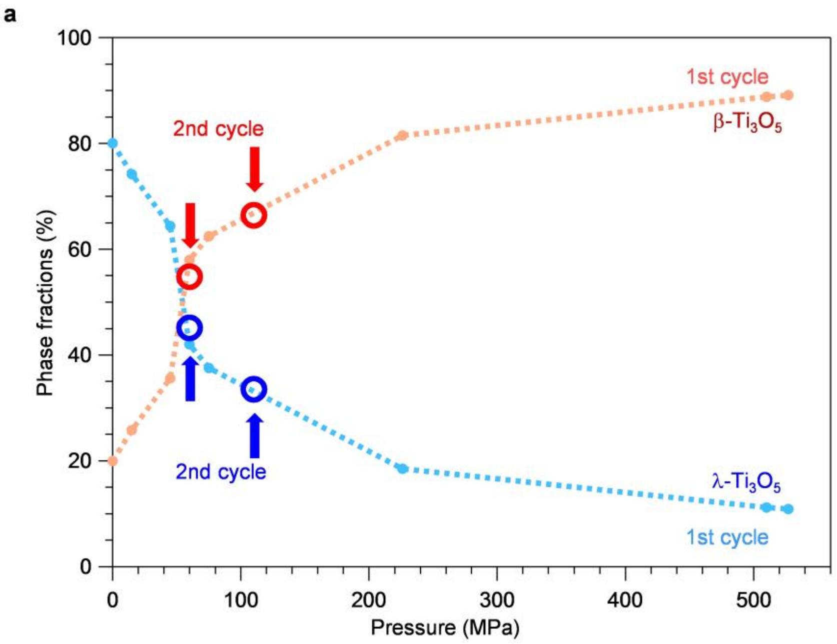

Supplementary Figure 6. Repeatability of the pressure-induced phase transition. The repeatability of the pressure-induced phase transition from stripe-type-λ-Ti_3O_5 to β-Ti_3O_5 was confirmed as follows: pressure application to the as-prepared sample at room temperature (1st cycle, as already shown in Fig. 1e) → heating to 620 K → cooling to room temperature → pressure application again (2nd cycle). The phase fractions of λ-Ti_3O_5 and β-Ti_3O_5 in the 1st cycle is shown with light blue and orange dotted lines, respectively. The 2nd cycle was carried out for the pressures of 60 MPa and 110 MPa, and the phase fractions of λ-Ti_3O_5 and β-Ti_3O_5 are shown with blue and red open circles, respectively.

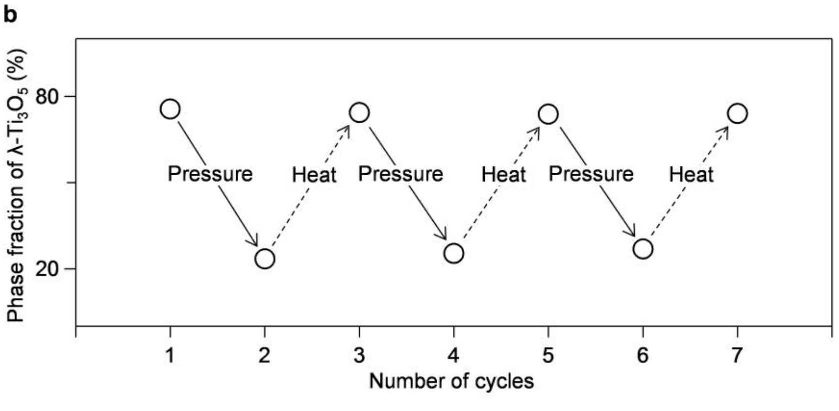

Supplementary Figure 7. Repeatability of the pressure-induced phase transition. In the pressure application process (solid lines), 510 MPa was applied at room temperature, and the phase fractions were obtained from the XRPD data of the pressure released sample. In the annealing process (dotted lines), the temperature was swept 300 K → 620 K → 300 K, and the phase fractions were obtained from the XRPD data at room temperature. The material transformed λ-Ti_3O_5 → β-Ti_3O_5 → λ-Ti_3O_5 .... , showing good repeatability of reversible pressure-and-heat phase transition.

a λ-Ti_3O_5

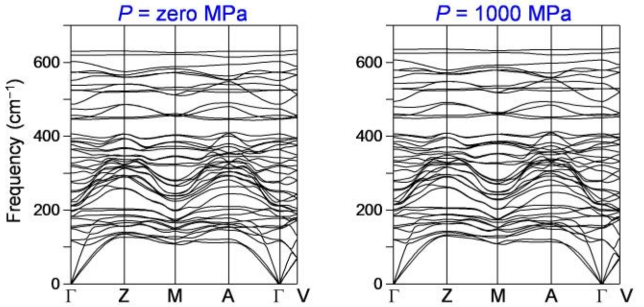

Frequency (cm^{-1})	Irreducible representation
116.6	A_u
119.7	B_u
149.4	B_g
152.0	A_g
155.9	A_g
176.8	B_g
182.7	A_u
183.3	A_g
196.5	B_g
198.9	B_g
211.1	A_u
212.0	A_g
212.8	B_u
224.0	B_g
225.6	B_u
248.6	B_u
252.3	A_u
274.7	B_u
277.6	A_g
300.5	A_g
318.5	B_u
322.6	B_u
327.1	A_g
342.1	A_g
364.2	A_g
364.3	B_u
376.9	B_u
383.7	A_g
387.5	A_g
393.6	B_u
405.9	A_g
445.8	B_u
448.2	A_u
456.7	A_g
474.2	B_g
486.3	A_u
524.8	B_u
525.4	A_g
538.1	A_u
551.1	B_g
572.4	B_g
573.8	B_u
602.5	A_g
619.5	B_u
630.2	A_g

Frequency (cm^{-1})	Irreducible representation
119.5	A_u
119.9	B_u
150.8	B_g
152.1	A_g
157.2	A_g
183.5	A_u
184.3	B_g
187.7	A_g
197.9	B_g
201.3	B_g
214.4	A_g
215.8	A_u
215.9	B_u
225.4	B_u
229.3	B_g
252.9	B_u
256.2	A_u
277.5	A_g
280.3	B_u
305.9	A_g
321.5	B_u
329.4	A_g
333.9	B_u
343.3	A_g
364.9	B_u
370.1	A_g
379.9	B_u
384.8	A_g
396.7	A_g
401.0	B_u
406.5	A_g
449.9	B_u
455.4	A_u
460.1	A_g
479.8	B_g
494.2	A_u
528.9	A_g
528.9	B_u
543.4	A_u
558.9	B_g
578.6	B_u
578.9	B_g
606.5	A_g
625.3	B_u
635.2	A_g

b β-Ti_3O_5

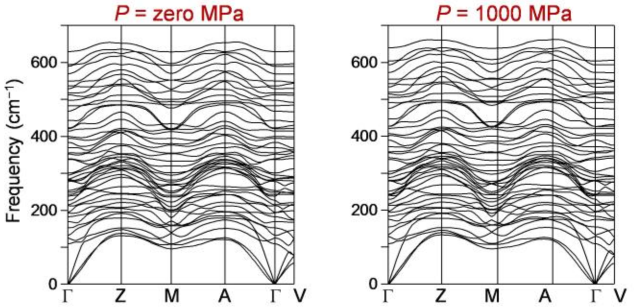

Frequency (cm^{-1})	Irreducible representation
109.2	B_g
124.9	A_g
131.7	A_u
152.2	B_u
169.8	A_g
182.4	B_g
193.6	A_g
194.1	A_u
212.5	B_u
214.7	B_g
226.7	B_u
238.1	A_g
242.9	A_u
248.6	B_g
253.0	A_g
278.1	B_u
281.3	A_u
284.5	B_g
284.6	A_g
295.1	B_u
300.8	A_g
338.0	A_g
339.3	B_u
352.0	B_u
355.1	A_g
369.5	B_u
384.0	A_g
394.8	B_u
399.9	A_g
414.3	B_g
420.6	A_u
422.7	B_u
443.2	A_g
468.3	A_u
483.1	B_u
498.0	A_g
512.8	B_u
517.8	A_g
528.6	B_g
536.5	A_g
550.0	B_u
569.2	A_u
589.8	B_g
594.4	B_u
629.6	A_g

Frequency (cm^{-1})	Irreducible representation
108.8	B_g
129.0	A_g
133.5	A_u
154.3	B_u
175.0	A_g
182.9	B_g
195.1	A_u
196.1	A_g
213.6	B_u
216.0	B_g
229.6	B_u
239.7	A_u
239.9	A_g
243.1	B_g
251.1	A_g
278.9	B_u
279.1	A_u
281.0	B_g
288.1	A_g
297.2	B_u
304.8	A_g
337.0	A_g
341.9	B_u
355.2	B_u
358.0	A_g
371.9	B_u
389.7	A_g
402.3	B_u
407.2	A_g
421.4	B_g
426.5	B_u
426.6	A_u
447.8	A_g
475.8	A_u
491.0	B_u
505.1	A_g
514.5	B_u
519.4	A_g
533.0	B_g
542.6	A_g
554.7	B_u
572.6	A_u
593.5	B_g
604.3	B_u
639.3	A_g

Supplementary Figure 8. Phonon dispersion and phonon frequencies obtained by first-principles calculation.
(**a**) Phonon dispersion and list of optical phonon frequencies at the Brillouin zone center, Γ point, for each of the phonon dispersions of λ-Ti_3O_5 calculated under zero MPa (≈ atmospheric pressure = 0.1 MPa) (left) and 1000 MPa (right).
(**b**) Phonon dispersion and list of phonon frequencies at the Brillouin zone center, Γ point, for each of the phonon dispersions of β-Ti_3O_5 calculated under zero MPa (left) and 1000 MPa (right). The lists show the optical phonon modes, excluding the acoustic phonon modes.

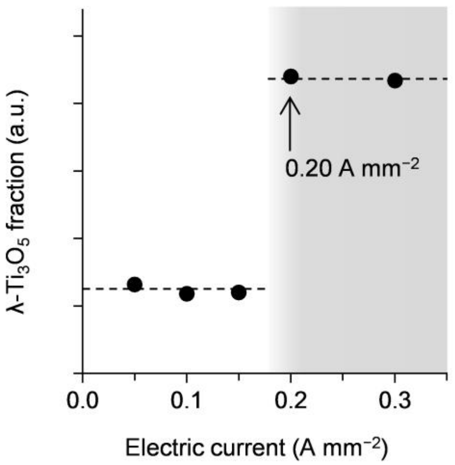

Supplementary Figure 9. The threshold current value of current-induced phase transition from pressure- produced β-Ti_3O_5 to λ-Ti_3O_5. The electric current dependence of λ-Ti_3O_5 phase fraction after flowing electric current was measured as follows: stainless electrodes were attached to the Pt-deposited β-Ti_3O_5 pellet by Ag paste, and electric current of 0.05, 0.10, 0.15, 0.20, and 0.30 A mm^{-2} were flowed at 298 K. Then, the pellet samples were grinded for the XRPD measurement. The dotted lines are for eye guide.

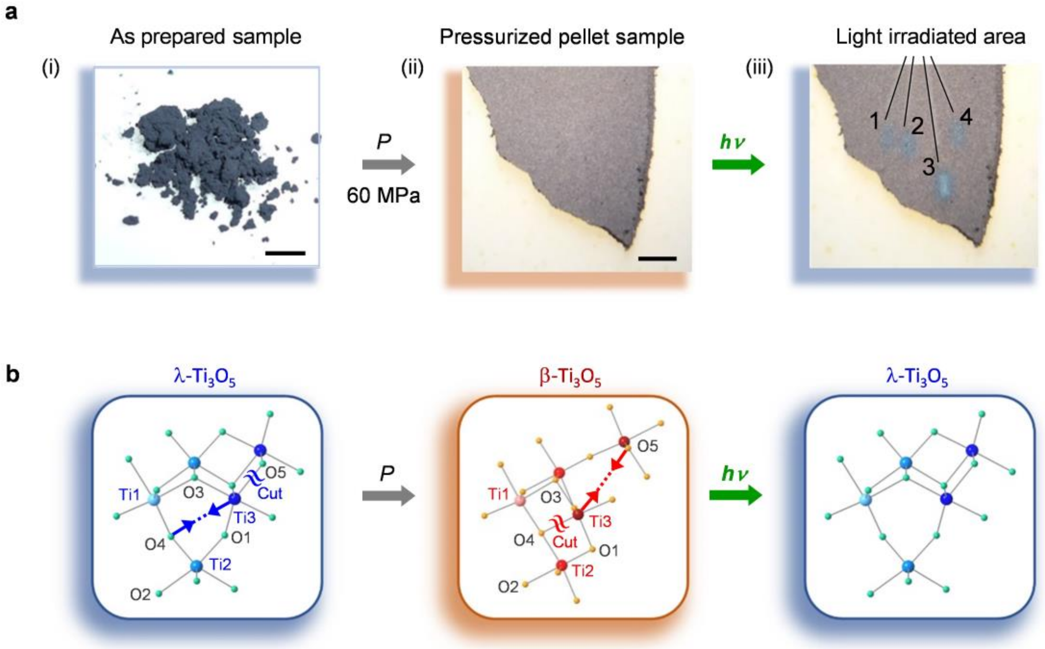

Supplementary Figure 10. Reversible pressure-and-light-induced phase transition between λ-Ti_3O_5 and β-Ti_3O_5. (**a**) Photographs of the as-prepared Ti_3O_5 (i), after applying pressure (P = 60 MPa) (ii), and after irradiating with 410-nm laser lights (5.2×10^{-3} mW μm^{-2}) (iii). The light irradiation was performed several times (light irradiation time: 3 sec at area 1, 5 sec at area 2, 20 sec at area 3, 15 sec at area 4). The scale bars in (i) and (ii) indicate 2 mm and 200 μm, respectively. (**b**) Schematic illustrations of partial crystal structure of λ-Ti_3O_5 (blue frame) and β-Ti_3O_5 (red frame). Blue arrows and wavy lines indicate the formation of the Ti(3)−O(4) bond and the breaking of the Ti(3)−O(5) bond, respectively, in the pressure-induced phase transition. Red arrows and wavy lines indicate the formation of Ti(3)−O(5) bond and the breaking of the Ti(3)−O(4) bond, respectively, in the light-induced phase transition.

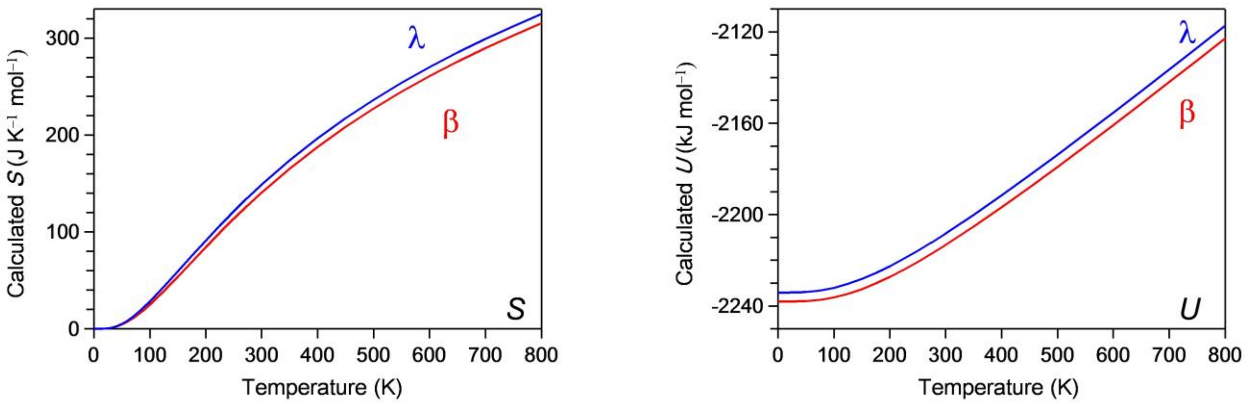

Supplementary Figure 11. Entropies and internal energies versus temperature curve of λ-Ti_3O_5 and β-Ti_3O_5. Entropies (S) and internal energies (U) versus temperature curve of λ-Ti_3O_5 (blue line) and β-Ti_3O_5 (red line) obtained from first-principles phonon mode calculations.

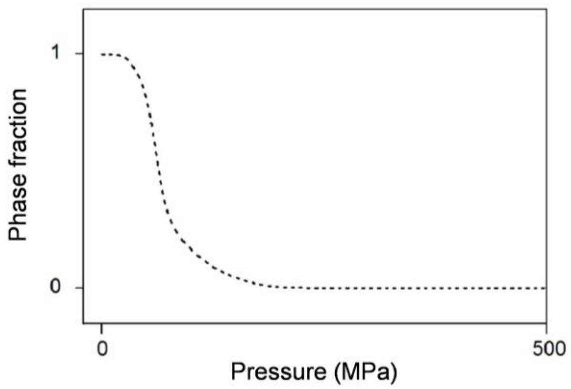

Supplementary Figure 12. Analysis of the gradual change of the pressure-induced phase transition. The observed x versus P plot is somewhat gradual compared to the calculated result in Fig. 5c. This is explained by the distribution in the transition pressure of the Slichter and Drickamer model, which may be due to the crystal size distribution. The gradual pressure-induced phase transition was calculated with a logarithmic normal distribution on the transition pressure.

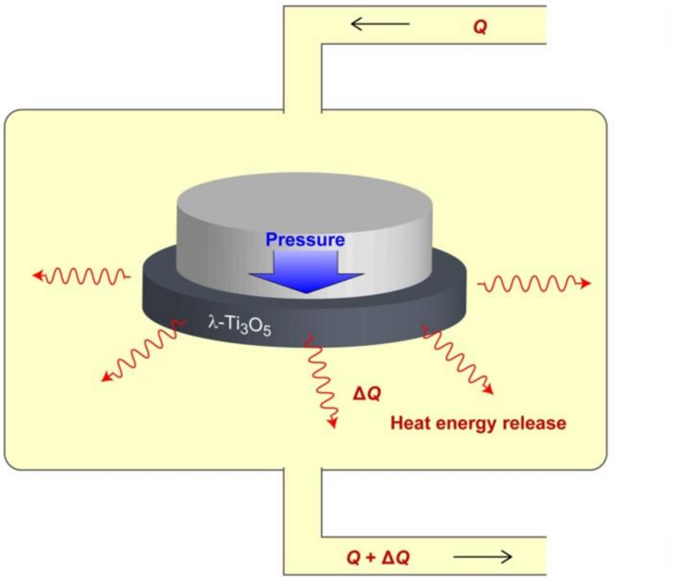

Supplementary Figure 13. Schematic illustration of a heat energy release system using λ-Ti_3O_5. Thermal energy can be stocked by the β-Ti_3O_5 → λ-Ti_3O_5 phase transition due to heat from blast furnace, or sunlight. Then, the stocked heat energy is released on demand by the λ-Ti_3O_5 → β-Ti_3O_5 phase transition upon application of pressure.

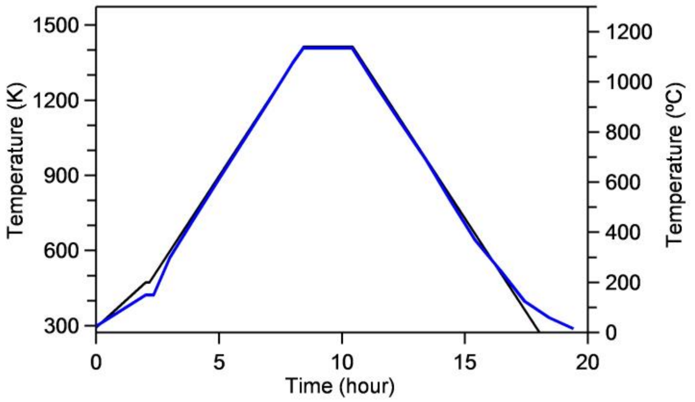

Supplementary Figure 14. Temperature chart in the sintering process of stripe-type-λ-Ti_3O_5. Black line is the programmed process, and blue line is the actual process.

a λ-Ti_3O_5

Temperature (K)	Entropy (*S*) ($J\ K^{-1}\ mol^{-1}$)	Internal energy (*U*) ($kJ\ mol^{-1}$)
0	0.000	-2234.064
1	0.000	-2234.064
2	0.000	-2234.064
3	0.000	-2234.064
4	0.000	-2234.064
5	0.000	-2234.064
10	0.002	-2234.064
15	0.030	-2234.064
20	0.145	-2234.062
30	0.868	-2234.043
40	2.490	-2233.986
50	5.068	-2233.869
60	8.506	-2233.679
70	12.669	-2233.408
80	17.424	-2233.051
90	22.656	-2232.606
100	28.263	-2232.073
125	43.380	-2230.370
150	59.302	-2228.180
175	75.374	-2225.568
200	91.199	-2222.602
225	106.550	-2219.341
250	121.310	-2215.837
273	134.325	-2212.434
275	135.430	-2212.131
298	147.847	-2208.575
300	148.901	-2208.260
350	173.966	-2200.124
400	196.735	-2191.595
450	217.492	-2182.781
500	236.505	-2173.756

b β-Ti_3O_5

Temperature (K)	Entropy (*S*) ($J\ K^{-1}\ mol^{-1}$)	Internal energy (*U*) ($kJ\ mol^{-1}$)
0	0.000	-2238.026
1	0.000	-2238.026
2	0.000	-2238.026
3	0.000	-2238.026
4	0.000	-2238.026
5	0.000	-2238.026
10	0.003	-2238.026
15	0.037	-2238.025
20	0.151	-2238.023
30	0.803	-2238.007
40	2.178	-2237.958
50	4.333	-2237.860
60	7.239	-2237.700
70	10.825	-2237.466
80	15.002	-2237.152
90	19.675	-2236.755
100	24.754	-2236.272
125	38.718	-2234.698
150	53.716	-2232.634
175	69.059	-2230.141
200	84.309	-2227.282
225	99.205	-2224.118
250	113.602	-2220.699
273	126.346	-2217.368
275	127.430	-2217.071
298	139.627	-2213.577
300	140.664	-2213.267
350	165.372	-2205.247
400	187.893	-2196.810
450	208.470	-2188.073
500	227.350	-2179.111

Table 1. Thermodynamic parameters from phonon mode calculations. Entropies (*S*) and internal energies (*U*) of (**a**) λ-Ti_3O_5 and (**b**) β-Ti_3O_5.

a

Temperature (K)	ΔU(1000 MPa) − ΔU(0 MPa) (kJ mol^{-1})	ΔU(60 MPa) − ΔU(0 MPa) (kJ mol^{-1})
0	0.136	0.008
1	0.136	0.008
2	0.136	0.008
3	0.136	0.008
4	0.136	0.008
5	0.136	0.008
10	0.136	0.008
15	0.136	0.008
20	0.136	0.008
30	0.136	0.008
40	0.134	0.008
50	0.130	0.008
60	0.124	0.007
70	0.118	0.007
80	0.110	0.007
90	0.103	0.006
100	0.095	0.006
125	0.077	0.005
150	0.063	0.004
175	0.051	0.003
200	0.041	0.002
225	0.034	0.002
250	0.028	0.002
273	0.023	0.001
275	0.023	0.001
298	0.019	0.001
300	0.019	0.001
350	0.013	0.001
400	0.008	0.001
450	0.005	0.000
500	0.003	0.000

b

Temperature (K)	ΔS(1000 MPa) − ΔS(0 MPa) (J K^{-1} mol^{-1})	ΔS(60 MPa) − ΔS(0 MPa) (J K^{-1} mol^{-1})
0	0.000	0.000
1	0.000	0.000
2	0.000	0.000
3	0.000	0.000
4	0.000	0.000
5	0.000	0.000
10	0.001	0.000
15	0.002	0.000
20	0.003	0.000
30	-0.015	-0.001
40	-0.070	-0.004
50	-0.156	-0.009
60	-0.256	-0.015
70	-0.359	-0.022
80	-0.458	-0.027
90	-0.548	-0.033
100	-0.628	-0.038
125	-0.786	-0.047
150	-0.893	-0.054
175	-0.967	-0.058
200	-1.017	-0.061
225	-1.053	-0.063
250	-1.078	-0.065
273	-1.095	-0.066
275	-1.096	-0.066
298	-1.109	-0.067
300	-1.110	-0.067
350	-1.129	-0.068
400	-1.141	-0.068
450	-1.149	-0.069
500	-1.155	-0.069

ΔU(60 MPa) is calculated by assuming that ΔU changes linearly between ΔU(0 MPa) and ΔU(1000 MPa), and ΔS(60 MPa) is calculated by assuming that ΔS changes linearly between ΔS(0 MPa) and ΔS(1000 MPa).

Supplementary Table 2. Pressure-induced change on internal energy and entropy calculated by first-principles phonon mode calculations. (**a**) Pressure-induced change on ΔU (= $U_\lambda - U_\beta$) between 1000 MPa and 0 MPa, i.e., ΔU(1000 MPa) − ΔU(0 MPa), and between 60 MPa and 0 MPa, i.e., ΔU(60 MPa) − ΔU(0 MPa). (**b**) Pressure-induced change of ΔS (= $S_\lambda - S_\beta$) between 1000 MPa and 0 MPa, i.e., ΔS(1000 MPa) − ΔS(0 MPa), and between 60 MPa and 0 MPa, i.e.,
ΔS(60 MPa) − ΔS(0 MPa).
